# 4-(4-Bromo­phen­yl)-8-methyl-2-oxo-1,2,3,4,4a,5,6,7-octa­hydro­quinoline-3-carbonitrile

**DOI:** 10.1107/S1600536812029820

**Published:** 2012-07-07

**Authors:** Abdullah M. Asiri, Hassan M. Faidallah, Seik Weng Ng, Edward R. T. Tiekink

**Affiliations:** a Center of Excellence for Advanced Materials Research (CEAMR), King Abdulaziz University, PO Box 80203, Jeddah 21589, Saudi Arabia; b Chemistry Department, Faculty of Science, King Abdulaziz University, PO Box 80203, Jeddah 21589, Saudi Arabia; c Department of Chemistry, University of Malaya, 50603 Kuala Lumpur, Malaysia

## Abstract

In the title compound, C_17_H_17_BrN_2_O, the N-containing ring adopts an envelope conformation with the C atom carrying the phenyl ring displaced by −0.531 (9) Å from the plane defined by the remaining five atoms (r.m.s. deviation = 0.0099 Å). The benzene ring is almost orthogonal to the ring to which it is attached, the C_CN_—C—C_Ph_—C_Ph_ torsion angle being −101.3 (7)°. The cyclo­hexene ring is disordered over two conformations in a statistical ratio. The most prominent inter­actions in the crystal are pairs of N—H⋯O hydrogen bonds between inversion-related mol­ecules. The resulting dimers are linked into a three-dimensional architecture by C—H⋯N, C—H⋯Br and C—H⋯π inter­actions.

## Related literature

For background to the cardiotonic and anti-inflammatory properties of octa­hydro­quinoline-3-carbonitrile derivatives, see: Behit & Baraka (2005 ▶); Girgis *et al.* (2007 ▶). For a related structure, see: Asiri *et al.* (2012 ▶). For additional conformational analysis, see: Cremer & Pople (1975 ▶).
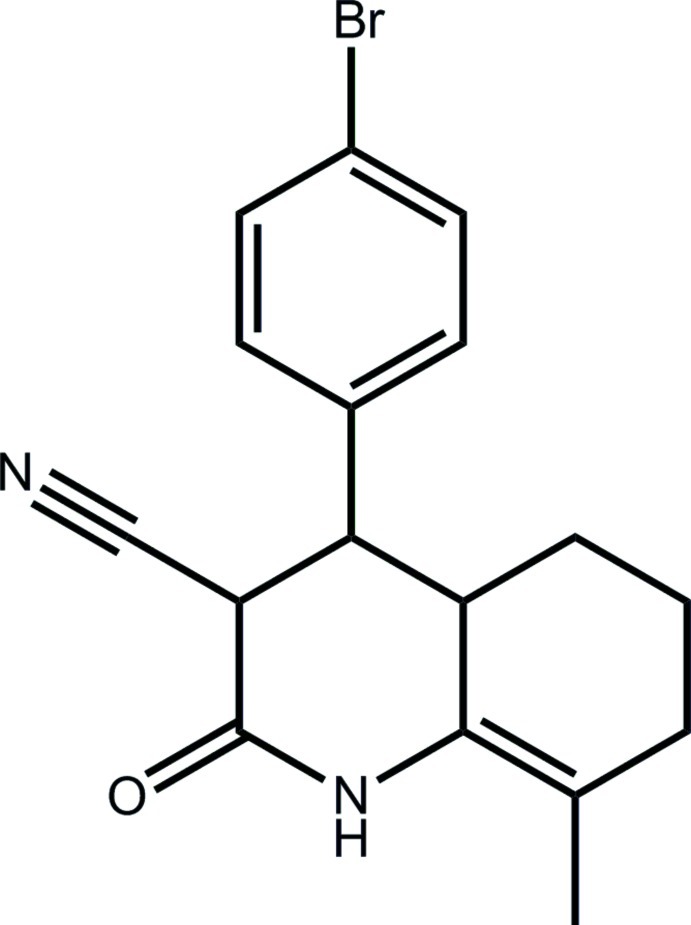


## Experimental

### 

#### Crystal data

C_17_H_17_BrN_2_O
*M* *_r_* = 345.24
Monoclinic, 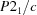

*a* = 11.1959 (10) Å
*b* = 7.5902 (6) Å
*c* = 18.3886 (12) Å
β = 100.453 (8)°
*V* = 1536.7 (2) Å^3^
*Z* = 4
Mo *K*α radiation
μ = 2.68 mm^−1^
*T* = 100 K
0.40 × 0.20 × 0.02 mm

#### Data collection

Agilent SuperNova Dual diffractometer with an Atlas detector
Absorption correction: multi-scan (*CrysAlis PRO*; Agilent, 2012 ▶) *T*_min_ = 0.581, *T*_max_ = 1.000
9883 measured reflections
3549 independent reflections
2296 reflections with *I* > 2σ(*I*)
*R*_int_ = 0.052

#### Refinement

*R*[*F* ^2^ > 2σ(*F* ^2^)] = 0.063
*wR*(*F* ^2^) = 0.173
*S* = 1.02
3549 reflections
199 parameters
22 restraints
H-atom parameters constrained
Δρ_max_ = 0.87 e Å^−3^
Δρ_min_ = −0.44 e Å^−3^

### 

Data collection: *CrysAlis PRO* (Agilent, 2012 ▶); cell refinement: *CrysAlis PRO*; data reduction: *CrysAlis PRO*; program(s) used to solve structure: *SHELXS97* (Sheldrick, 2008 ▶); program(s) used to refine structure: *SHELXL97* (Sheldrick, 2008 ▶); molecular graphics: *ORTEP-3 for Windows* (Farrugia, 1997 ▶) and *DIAMOND* (Brandenburg, 2006 ▶); software used to prepare material for publication: *publCIF* (Westrip, 2010 ▶).

## Supplementary Material

Crystal structure: contains datablock(s) global, I. DOI: 10.1107/S1600536812029820/hb6875sup1.cif

Structure factors: contains datablock(s) I. DOI: 10.1107/S1600536812029820/hb6875Isup2.hkl

Supplementary material file. DOI: 10.1107/S1600536812029820/hb6875Isup3.cml

Additional supplementary materials:  crystallographic information; 3D view; checkCIF report

## Figures and Tables

**Table 1:** Hydrogen-bond geometry (Å, °) *Cg*1 is the centroid of the C12–C17 benzene ring.

*D*—H⋯*A*	*D*—H	H⋯*A*	*D*⋯*A*	*D*—H⋯*A*
N1—H1*n*⋯O1^i^	0.88	2.08	2.921 (5)	161
C1—H1*A*⋯N2^ii^	0.98	2.57	3.442 (8)	148
C13—H13⋯Br1^iii^	0.95	2.86	3.811 (6)	174
C1—H1*B*⋯*Cg*1^iv^	0.98	2.78	3.590 (6)	141

Symmetry codes: (i) 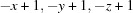; (ii) 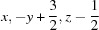; (iii) 
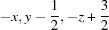
; (iv) 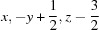.

**Fig. 1.**
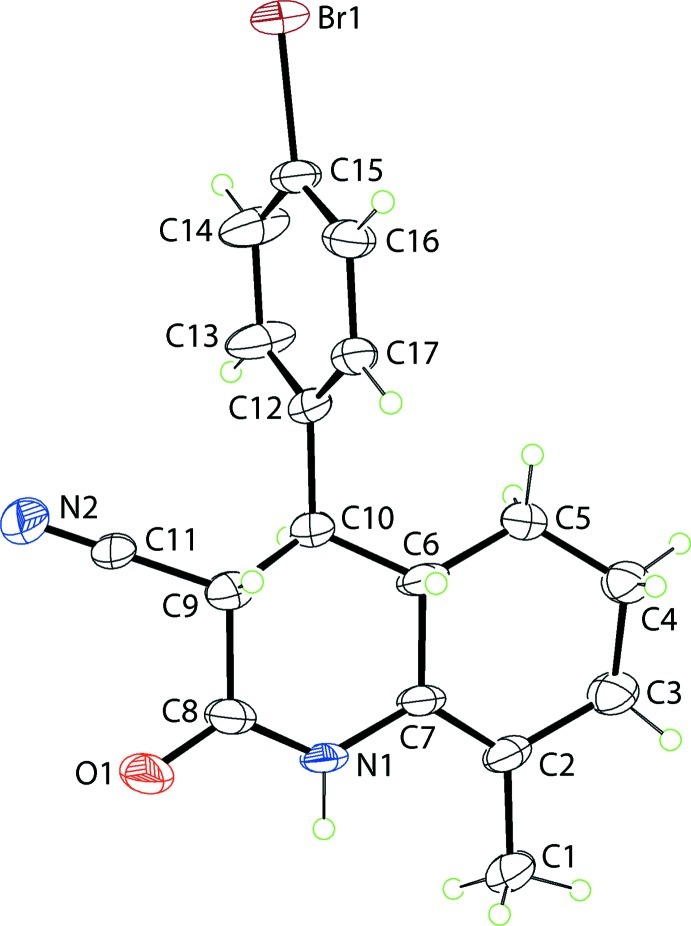
The molecular structure of (I) showing displacement ellipsoids at the 35% probability level.

**Fig. 2.**
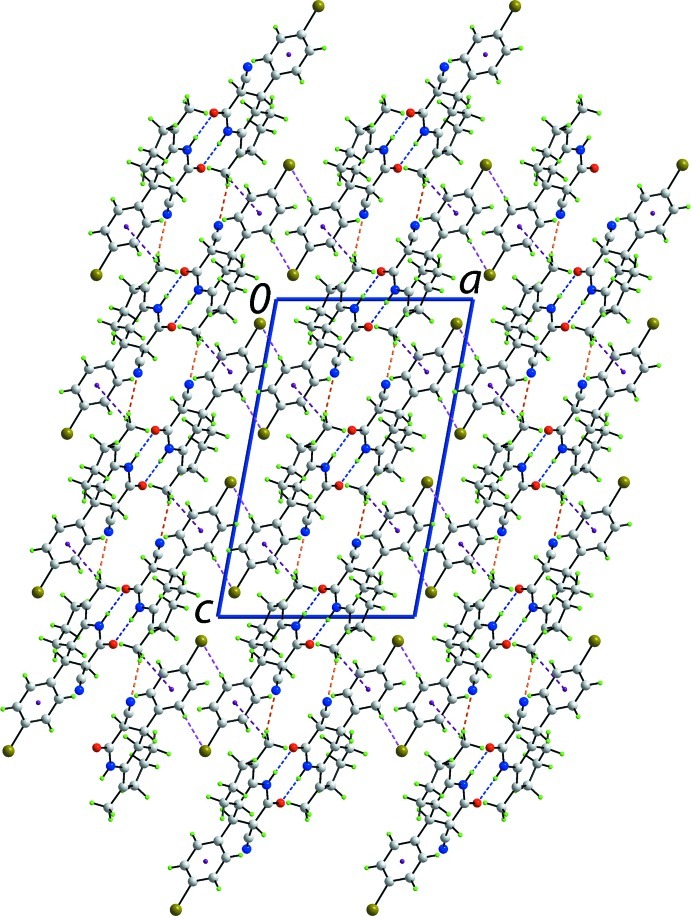
A view in projection down the b axis of the unit-cell contents of (I), The N—H···O, C—H···N, C—H···Br and and C—H···.π interactions are shown as blue, orange, pink and purple dashed lines, respectively.

## supplementary crystallographic information

### Comment

In continuation of our structural studies on octahydroquinoline-3-carbonitrile derivatives (Asiri *et al.*, 2012), the crystal and molecular structure of the title compound, (I), was investigated. Interest in this class of compound stems from their demonstrated cardiotonic and anti-inflammatory properties (Behit & Baraka, 2005; Girgis *et al.*, 2007).

In (I), Fig. 1, the N1-containing ring of the fused octahydroquinoline fused-ring system, adopts an envelope conformation with the C10 atom, carrying the phenyl ring, lying -0.531 (9)° out of the plane defined by the remaining five atoms [r.m.s. deviation = 0.0099 Å]. Owing to two conformations of equal weight, the assignment of conformation of the C_6_ ring is somewhat problematic but a conformational analysis indicates an intermediate conformation between screw-boat and half-chair (Cremer & Pople, 1975). The phenyl ring is almost orthogonal to the ring to which it is attached with the C9—C10—C12—C13 torsion angle being -101.3 (7)°.

The most prominent interactions in the crystal packing is a pair of N—H···O hydrogen bonds between inversion related molecules leading to an eight-membered {···HNCO}_2_ synthon, Table 1. These are linked into a three-dimensional architecture by C—H···N, C—H···Br and C—H···π interactions, Fig. 2 and Table 1.

### Experimental

A mixture of the *p*-bromobenzaldehyde (1.4 g, 0.01 *M*), 2-methylcyclohexanone (1.2 g, 0.01 *M*), ethyl cyanoacetate (1.1 g, 0.01 *M*) and ammonium acetate (6.2 g, 0.0 8*M*) in absolute ethanol (50 ml) was refluxed for 6 h. The reaction mixture was allowed to cool, the formed precipitate was filtered, washed with water, dried and recrystallized from its ethanol solution as light yellow plates. *M*.pt: 511–513 K. Yield: 78%.

### Refinement

Carbon-bound H-atoms were placed in calculated positions [C—H = 0.95–0.99 Å, *U*_iso_(H) = 1.2–1.5*U*_eq_(C)] and were included in the refinement in the riding model approximation. The N-bound H-atom was treated similarly with N—H = 0.88 Å and with *U*_iso_(H) = 1.2*U*_eq_(N). A part of the cyclohexene ring is disordered over two positions in an assumed 1:1 ratio. Pairs of 1,2-related distances were restrained to within 0.01 Å of each other. The anisotropic displacement parameters, restrained to be nearly isotropic, of the primed atoms were set to those of the unprimed ones. The amino H-atom is less than 2 Å from a methyl H-atom. As the methyl group was refined as a disordered methyl group, the short H1···H1d contact of 1.84 Å is an artifact.

### Crystal data

**Table:**

C_17_H_17_BrN_2_O	*F*(000) = 704
*M**_r_* = 345.24	*D*_x_ = 1.492 Mg m^−^^3^
Monoclinic, *P*2_1_/*c*	Mo *K*α radiation, λ = 0.71073 Å
Hall symbol: -P 2ybc	Cell parameters from 2464 reflections
*a* = 11.1959 (10) Å	θ = 2.3–27.5°
*b* = 7.5902 (6) Å	µ = 2.68 mm^−^^1^
*c* = 18.3886 (12) Å	*T* = 100 K
β = 100.453 (8)°	Plate, light-yellow
*V* = 1536.7 (2) Å^3^	0.40 × 0.20 × 0.02 mm
*Z* = 4	

### Data collection

**Table:**

Agilent SuperNova Dual diffractometer with an Atlas detector	3549 independent reflections
Radiation source: SuperNova (Mo) X-ray Source	2296 reflections with *I* > 2σ(*I*)
Mirror monochromator	*R*_int_ = 0.052
Detector resolution: 10.4041 pixels mm^-1^	θ_max_ = 27.6°, θ_min_ = 2.6°
ω scan	*h* = −14→9
Absorption correction: multi-scan (*CrysAlis PRO*; Agilent, 2012)	*k* = −9→9
*T*_min_ = 0.581, *T*_max_ = 1.000	*l* = −22→23
9883 measured reflections	

### Refinement

**Table:**

Refinement on *F*^2^	Primary atom site location: structure-invariant direct methods
Least-squares matrix: full	Secondary atom site location: difference Fourier map
*R*[*F*^2^ > 2σ(*F*^2^)] = 0.063	Hydrogen site location: inferred from neighbouring sites
*wR*(*F*^2^) = 0.173	H-atom parameters constrained
*S* = 1.02	*w* = 1/[σ^2^(*F*_o_^2^) + (0.0744*P*)^2^ + 2.0179*P*] where *P* = (*F*_o_^2^ + 2*F*_c_^2^)/3
3549 reflections	(Δ/σ)_max_ < 0.001
199 parameters	Δρ_max_ = 0.87 e Å^−^^3^
22 restraints	Δρ_min_ = −0.44 e Å^−^^3^

### Special details

**Table:**

Geometry. All e.s.d.'s (except the e.s.d. in the dihedral angle between two l.s. planes) are estimated using the full covariance matrix. The cell e.s.d.'s are taken into account individually in the estimation of e.s.d.'s in distances, angles and torsion angles; correlations between e.s.d.'s in cell parameters are only used when they are defined by crystal symmetry. An approximate (isotropic) treatment of cell e.s.d.'s is used for estimating e.s.d.'s involving l.s. planes.
Refinement. Refinement of *F*^2^ against ALL reflections. The weighted *R*-factor *wR* and goodness of fit *S* are based on *F*^2^, conventional *R*-factors *R* are based on *F*, with *F* set to zero for negative *F*^2^. The threshold expression of *F*^2^ > σ(*F*^2^) is used only for calculating *R*-factors(gt) *etc*. and is not relevant to the choice of reflections for refinement. *R*-factors based on *F*^2^ are statistically about twice as large as those based on *F*, and *R*- factors based on ALL data will be even larger.

### Fractional atomic coordinates and isotropic or equivalent isotropic displacement parameters (Å^2^)

**Table:**

	*x*	*y*	*z*	*U*_iso_*/*U*_eq_	Occ. (<1)
Br1	0.06147 (5)	0.90018 (7)	0.92341 (3)	0.0586 (2)	
O1	0.4933 (5)	0.4661 (6)	0.5872 (2)	0.0912 (16)	
N1	0.4052 (4)	0.7076 (6)	0.5296 (2)	0.0576 (12)	
H1n	0.4373	0.6809	0.4907	0.069*	
N2	0.3645 (6)	0.3614 (6)	0.7319 (3)	0.0836 (18)	
C1	0.3778 (6)	0.9254 (7)	0.3984 (3)	0.0579 (14)	
H1A	0.3554	1.0180	0.3613	0.087*	0.50
H1B	0.3492	0.8112	0.3771	0.087*	0.50
H1C	0.4663	0.9223	0.4136	0.087*	0.50
H1D	0.4252	0.8163	0.4067	0.087*	0.50
H1E	0.4314	1.0231	0.3909	0.087*	0.50
H1F	0.3143	0.9120	0.3544	0.087*	0.50
C2	0.3211 (5)	0.9625 (7)	0.4635 (3)	0.0585 (14)	
C3	0.2661 (15)	1.1429 (16)	0.4681 (7)	0.061 (4)	0.50
H3A	0.3222	1.2288	0.4516	0.073*	0.50
H3B	0.1900	1.1461	0.4311	0.073*	0.50
C4	0.2372 (13)	1.2098 (17)	0.5380 (6)	0.063 (3)	0.50
H4A	0.3078	1.2763	0.5647	0.075*	0.50
H4B	0.1679	1.2925	0.5268	0.075*	0.50
C5	0.2067 (11)	1.0704 (13)	0.5856 (8)	0.055 (4)	0.50
H5A	0.1212	1.0358	0.5676	0.066*	0.50
H5B	0.2114	1.1197	0.6359	0.066*	0.50
C3'	0.2305 (15)	1.1102 (18)	0.4484 (7)	0.061 (4)	0.50
H3C	0.2712	1.2160	0.4330	0.073*	0.50
H3D	0.1650	1.0762	0.4071	0.073*	0.50
C4'	0.1773 (13)	1.1532 (17)	0.5133 (7)	0.063 (3)	0.50
H4C	0.2227	1.2550	0.5382	0.075*	0.50
H4D	0.0930	1.1935	0.4952	0.075*	0.50
C5'	0.1727 (10)	1.0196 (15)	0.5692 (8)	0.055 (4)	0.50
H5C	0.1035	0.9404	0.5508	0.066*	0.50
H5D	0.1547	1.0790	0.6139	0.066*	0.50
C6	0.2820 (6)	0.9091 (8)	0.5917 (3)	0.075 (2)	
H6	0.3575	0.9591	0.6222	0.090*	0.50
H6'	0.3437	0.9871	0.6221	0.090*	0.50
C7	0.3338 (5)	0.8612 (7)	0.5242 (3)	0.0543 (14)	
C8	0.4298 (6)	0.5976 (8)	0.5872 (3)	0.0675 (18)	
C9	0.3741 (6)	0.6431 (8)	0.6547 (3)	0.0667 (17)	
H9	0.4373	0.7148	0.6872	0.080*	
C10	0.2671 (5)	0.7576 (6)	0.6390 (2)	0.0457 (11)	
H10	0.2039	0.6833	0.6079	0.055*	
C11	0.3637 (5)	0.4810 (7)	0.6963 (2)	0.0522 (13)	
C12	0.2137 (4)	0.7995 (6)	0.7068 (2)	0.0423 (11)	
C13	0.1125 (6)	0.7135 (9)	0.7198 (3)	0.0698 (18)	
H13	0.0740	0.6294	0.6850	0.084*	
C14	0.0646 (5)	0.7472 (9)	0.7836 (3)	0.0704 (18)	
H14	−0.0070	0.6887	0.7914	0.085*	
C15	0.1213 (5)	0.8642 (6)	0.8342 (2)	0.0447 (11)	
C16	0.2224 (5)	0.9533 (7)	0.8224 (3)	0.0507 (12)	
H16	0.2602	1.0378	0.8573	0.061*	
C17	0.2689 (5)	0.9188 (6)	0.7589 (3)	0.0476 (12)	
H17	0.3401	0.9784	0.7511	0.057*	

### Atomic displacement parameters (Å^2^)

**Table:**

	*U*^11^	*U*^22^	*U*^33^	*U*^12^	*U*^13^	*U*^23^
Br1	0.0719 (4)	0.0740 (4)	0.0378 (3)	0.0033 (3)	0.0312 (3)	−0.0034 (2)
O1	0.134 (4)	0.098 (3)	0.057 (2)	0.072 (3)	0.058 (3)	0.033 (2)
N1	0.072 (3)	0.068 (3)	0.044 (2)	0.031 (2)	0.041 (2)	0.020 (2)
N2	0.153 (6)	0.055 (3)	0.057 (3)	0.021 (3)	0.057 (3)	0.010 (2)
C1	0.076 (4)	0.061 (3)	0.043 (3)	−0.003 (3)	0.028 (3)	0.007 (2)
C2	0.066 (4)	0.068 (3)	0.050 (3)	0.016 (3)	0.035 (3)	0.022 (3)
C3	0.072 (8)	0.073 (5)	0.041 (6)	0.023 (5)	0.020 (5)	0.018 (4)
C4	0.069 (7)	0.066 (6)	0.061 (6)	0.030 (5)	0.032 (5)	0.021 (4)
C5	0.071 (6)	0.052 (6)	0.051 (6)	0.017 (5)	0.032 (5)	0.006 (5)
C3'	0.072 (8)	0.073 (5)	0.041 (6)	0.023 (5)	0.020 (5)	0.018 (4)
C4'	0.069 (7)	0.066 (6)	0.061 (6)	0.030 (5)	0.032 (5)	0.021 (4)
C5'	0.071 (6)	0.052 (6)	0.051 (6)	0.017 (5)	0.032 (5)	0.006 (5)
C6	0.090 (5)	0.090 (4)	0.062 (3)	0.047 (4)	0.058 (3)	0.040 (3)
C7	0.060 (3)	0.067 (3)	0.045 (3)	0.024 (3)	0.035 (2)	0.017 (2)
C8	0.085 (4)	0.077 (4)	0.051 (3)	0.042 (3)	0.041 (3)	0.019 (3)
C9	0.096 (5)	0.066 (3)	0.050 (3)	0.028 (3)	0.043 (3)	0.017 (3)
C10	0.064 (3)	0.044 (3)	0.034 (2)	0.004 (2)	0.023 (2)	0.000 (2)
C11	0.090 (4)	0.044 (3)	0.031 (2)	0.009 (3)	0.032 (2)	0.000 (2)
C12	0.057 (3)	0.043 (2)	0.032 (2)	−0.001 (2)	0.021 (2)	0.0019 (19)
C13	0.079 (4)	0.096 (4)	0.043 (3)	−0.039 (4)	0.034 (3)	−0.027 (3)
C14	0.070 (4)	0.100 (5)	0.049 (3)	−0.039 (3)	0.032 (3)	−0.020 (3)
C15	0.048 (3)	0.056 (3)	0.035 (2)	0.002 (2)	0.022 (2)	−0.002 (2)
C16	0.058 (3)	0.053 (3)	0.046 (3)	−0.001 (2)	0.024 (2)	−0.011 (2)
C17	0.059 (3)	0.041 (2)	0.051 (3)	−0.004 (2)	0.032 (2)	−0.003 (2)

### Geometric parameters (Å, º)

**Table:**

Br1—C15	1.900 (4)	C3'—H3D	0.9900
O1—C8	1.225 (6)	C4'—C5'	1.451 (10)
N1—C8	1.336 (6)	C4'—H4C	0.9900
N1—C7	1.407 (6)	C4'—H4D	0.9900
N1—H1n	0.8800	C5'—C6	1.480 (9)
N2—C11	1.118 (6)	C5'—H5C	0.9900
C1—C2	1.481 (7)	C5'—H5D	0.9900
C1—H1A	0.9800	C6—C10	1.469 (7)
C1—H1B	0.9800	C6—C7	1.507 (7)
C1—H1C	0.9800	C6—H6	1.0000
C1—H1D	0.9800	C6—H6'	1.0000
C1—H1E	0.9800	C8—C9	1.527 (7)
C1—H1F	0.9800	C9—C11	1.465 (7)
C2—C7	1.342 (7)	C9—C10	1.465 (7)
C2—C3'	1.503 (9)	C9—H9	1.0000
C2—C3	1.510 (9)	C10—C12	1.511 (6)
C3—C4	1.471 (10)	C10—H10	1.0000
C3—H3A	0.9900	C12—C13	1.366 (7)
C3—H3B	0.9900	C12—C17	1.380 (7)
C4—C5	1.454 (10)	C13—C14	1.398 (7)
C4—H4A	0.9900	C13—H13	0.9500
C4—H4B	0.9900	C14—C15	1.358 (7)
C5—C6	1.479 (9)	C14—H14	0.9500
C5—H5A	0.9900	C15—C16	1.369 (7)
C5—H5B	0.9900	C16—C17	1.387 (6)
C3'—C4'	1.464 (10)	C16—H16	0.9500
C3'—H3C	0.9900	C17—H17	0.9500
			
C8—N1—C7	127.3 (4)	H4C—C4'—H4D	107.0
C8—N1—H1n	116.3	C4'—C5'—C6	117.4 (10)
C7—N1—H1n	116.3	C4'—C5'—H5C	107.9
C2—C1—H1A	109.5	C6—C5'—H5C	107.9
C2—C1—H1B	109.5	C4'—C5'—H5D	107.9
H1A—C1—H1B	109.5	C6—C5'—H5D	107.9
C2—C1—H1C	109.5	H5C—C5'—H5D	107.2
H1A—C1—H1C	109.5	C10—C6—C5'	115.6 (7)
H1B—C1—H1C	109.5	C10—C6—C5	124.6 (6)
C2—C1—H1D	109.5	C5'—C6—C5	22.9 (9)
H1A—C1—H1D	141.1	C10—C6—C7	113.7 (5)
H1B—C1—H1D	56.3	C5'—C6—C7	109.2 (7)
H1C—C1—H1D	56.3	C5—C6—C7	115.9 (6)
C2—C1—H1E	109.5	C10—C6—H6	98.1
H1A—C1—H1E	56.3	C5'—C6—H6	120.9
H1B—C1—H1E	141.1	C5—C6—H6	98.1
H1C—C1—H1E	56.3	C7—C6—H6	98.1
H1D—C1—H1E	109.5	C10—C6—H6'	105.9
C2—C1—H1F	109.5	C5'—C6—H6'	105.9
H1A—C1—H1F	56.3	C7—C6—H6'	105.9
H1B—C1—H1F	56.3	C2—C7—N1	120.4 (4)
H1C—C1—H1F	141.1	C2—C7—C6	123.3 (5)
H1D—C1—H1F	109.5	N1—C7—C6	116.1 (4)
H1E—C1—H1F	109.5	O1—C8—N1	123.1 (5)
C7—C2—C1	124.6 (5)	O1—C8—C9	120.4 (5)
C7—C2—C3'	123.2 (7)	N1—C8—C9	116.6 (4)
C1—C2—C3'	111.5 (6)	C11—C9—C10	117.5 (5)
C7—C2—C3	117.1 (6)	C11—C9—C8	108.5 (4)
C1—C2—C3	117.2 (6)	C10—C9—C8	114.4 (4)
C4—C3—C2	121.3 (10)	C11—C9—H9	105.0
C4—C3—H3A	107.0	C10—C9—H9	105.0
C2—C3—H3A	107.0	C8—C9—H9	105.0
C4—C3—H3B	107.0	C9—C10—C6	113.8 (5)
C2—C3—H3B	107.0	C9—C10—C12	113.2 (4)
H3A—C3—H3B	106.7	C6—C10—C12	115.4 (4)
C5—C4—C3	112.8 (13)	C9—C10—H10	104.3
C5—C4—H4A	109.0	C6—C10—H10	104.3
C3—C4—H4A	109.0	C12—C10—H10	104.3
C5—C4—H4B	109.0	N2—C11—C9	174.1 (7)
C3—C4—H4B	109.0	C13—C12—C17	118.3 (4)
H4A—C4—H4B	107.8	C13—C12—C10	120.6 (4)
C4—C5—C6	117.1 (10)	C17—C12—C10	121.1 (4)
C4—C5—H5A	108.0	C12—C13—C14	121.0 (5)
C6—C5—H5A	108.0	C12—C13—H13	119.5
C4—C5—H5B	108.0	C14—C13—H13	119.5
C6—C5—H5B	108.0	C15—C14—C13	119.5 (5)
H5A—C5—H5B	107.3	C15—C14—H14	120.3
C4'—C3'—C2	112.1 (9)	C13—C14—H14	120.3
C4'—C3'—H3C	109.2	C14—C15—C16	120.7 (4)
C2—C3'—H3C	109.2	C14—C15—Br1	119.4 (4)
C4'—C3'—H3D	109.2	C16—C15—Br1	119.8 (4)
C2—C3'—H3D	109.2	C15—C16—C17	119.2 (5)
H3C—C3'—H3D	107.9	C15—C16—H16	120.4
C5'—C4'—C3'	119.5 (12)	C17—C16—H16	120.4
C5'—C4'—H4C	107.4	C12—C17—C16	121.3 (5)
C3'—C4'—H4C	107.4	C12—C17—H17	119.4
C5'—C4'—H4D	107.4	C16—C17—H17	119.4
C3'—C4'—H4D	107.4		
			
C7—C2—C3—C4	4.8 (19)	C7—N1—C8—O1	−179.9 (7)
C1—C2—C3—C4	−163.6 (12)	C7—N1—C8—C9	0.2 (10)
C3'—C2—C3—C4	117 (4)	O1—C8—C9—C11	−24.4 (9)
C2—C3—C4—C5	−30 (2)	N1—C8—C9—C11	155.5 (6)
C3—C4—C5—C6	42.1 (18)	O1—C8—C9—C10	−157.8 (7)
C7—C2—C3'—C4'	11.8 (19)	N1—C8—C9—C10	22.1 (9)
C1—C2—C3'—C4'	−177.7 (11)	C11—C9—C10—C6	−175.0 (5)
C3—C2—C3'—C4'	−67 (2)	C8—C9—C10—C6	−46.0 (8)
C2—C3'—C4'—C5'	−25 (2)	C11—C9—C10—C12	50.7 (7)
C3'—C4'—C5'—C6	43 (2)	C8—C9—C10—C12	179.7 (5)
C4'—C5'—C6—C10	−170.4 (10)	C5'—C6—C10—C9	174.9 (8)
C4'—C5'—C6—C5	71 (2)	C5—C6—C10—C9	−160.7 (9)
C4'—C5'—C6—C7	−40.8 (15)	C7—C6—C10—C9	47.5 (8)
C4—C5—C6—C10	177.9 (10)	C5'—C6—C10—C12	−51.8 (10)
C4—C5—C6—C5'	−109 (3)	C5—C6—C10—C12	−27.4 (12)
C4—C5—C6—C7	−30.9 (16)	C7—C6—C10—C12	−179.2 (5)
C1—C2—C7—N1	0.2 (10)	C9—C10—C12—C13	−101.3 (7)
C3'—C2—C7—N1	169.4 (10)	C6—C10—C12—C13	125.1 (6)
C3—C2—C7—N1	−167.4 (9)	C9—C10—C12—C17	75.4 (6)
C1—C2—C7—C6	175.6 (6)	C6—C10—C12—C17	−58.2 (7)
C3'—C2—C7—C6	−15.1 (13)	C17—C12—C13—C14	1.1 (9)
C3—C2—C7—C6	8.1 (12)	C10—C12—C13—C14	177.9 (6)
C8—N1—C7—C2	177.4 (6)	C12—C13—C14—C15	−1.6 (10)
C8—N1—C7—C6	1.7 (9)	C13—C14—C15—C16	2.0 (9)
C10—C6—C7—C2	158.9 (6)	C13—C14—C15—Br1	−176.5 (5)
C5'—C6—C7—C2	28.2 (11)	C14—C15—C16—C17	−2.0 (8)
C5—C6—C7—C2	4.5 (12)	Br1—C15—C16—C17	176.6 (4)
C10—C6—C7—N1	−25.4 (8)	C13—C12—C17—C16	−1.0 (8)
C5'—C6—C7—N1	−156.1 (7)	C10—C12—C17—C16	−177.8 (4)
C5—C6—C7—N1	−179.8 (8)	C15—C16—C17—C12	1.4 (8)

### Hydrogen-bond geometry (Å, º)

Cg1 is the centroid of the C12–C17 benzene ring.

**Table:**

*D*—H···*A*	*D*—H	H···*A*	*D*···*A*	*D*—H···*A*
N1—H1*n*···O1^i^	0.88	2.08	2.921 (5)	161
C1—H1*A*···N2^ii^	0.98	2.57	3.442 (8)	148
C13—H13···Br1^iii^	0.95	2.86	3.811 (6)	174
C1—H1*B*···*Cg*1^iv^	0.98	2.78	3.590 (6)	141

Symmetry codes: (i) −*x*+1, −*y*+1, −*z*+1; (ii) *x*, −*y*+3/2, *z*−1/2; (iii) −*x*, *y*−1/2, −*z*+3/2; (iv) *x*, −*y*+1/2, *z*−3/2.
